# Practical Prediction Model for Ovarian Insufficiency after Radiation

**DOI:** 10.1055/s-0042-1746199

**Published:** 2022-05-26

**Authors:** Gabriel Oliveira Bernardes Gil, Cassiano Asano, Warne Pedro de Andrade, Maria Luísa Braga Vieira Gil, Eduardo Batista Cândido, Marcos Regalin, Izabella Nobre Queiroz, Farley Soares Cantídio, Darly Gomes Soares Delfino, Agnaldo Lopes Silva-Filho

**Affiliations:** 1 Radiotherapy Department, Hospital Mater Dei, Belo Horizonte, MG, Brazil; 2 Gynecology Department, Universidade Estadual Paulista “Júlio de Mesquita Filho”, Botucatu, SP, Brazil; 3 Oncology Department, Grupo Oncoclínicas, Belo Horizonte, MG, Brazil; 4 Gynecology Department, Universidade Federal de Minas Gerais, Belo Horizonte, MG, Brazil; 5 Radiotherapy Department, Hospital da Baleia, Belo Horizonte, MG, Brazil; 6 Faculty of Medicine, Faculdade de Minas, Belo Horizonte, MG, Brazil

**Keywords:** radiotherapy, ovarian insufficiency, ovarian function, ovarian radiation, radioterapia, insuficiência ovariana, função ovariana, radiação ovariana

## Abstract

**Objective** The present study aimed to develop a useful mathematical model that predicts the age at which premature ovarian insufficiency might occur after teletherapy radiation. A diagnosis of premature or early menopause has physical and psychological consequences, so women may need support and long-term medical follow-up.

**Methods** To correlate ovarian radiation dose with ovarian function, we used the formula described by Wallace et al.: √g(z) = 10 ^(2-0,15z)^ , where “g(z)” and “z” represent oocyte survival rate and the radiation dose (in Gray), respectively. By simulating different ages and doses, we observed a pattern that could be used to simplify the relationship between radiation dose and remaining time of ovarian function.

**Results** We obtained a linear function between ovarian radiation dose and loss of ovarian function (LOF) that is the percentage of decrease in the time to the ovarian failure compared with the time expected for a woman at the same age without irradiation exposition. For patients < 40 years old and with ovarian radiation doses < 5 Gy, the equation LOF = 2.70 + (11.08 x Dose) can be applied to estimate the decrease in time to premature ovarian insufficiency.

**Conclusion** The present study reports a practicable theoretical method to estimate the loss of ovarian function. These findings can potentially improve the management and counseling of young women patients submitted to radiotherapy during their reproductive years.

## Resumo

**Objetivo** O presente estudo teve como objetivo desenvolver um modelo matemático útil que prediz a idade na qual a insuficiência ovariana prematura pode ocorrer após a radioterapia externa (teleterapia). O diagnóstico de menopausa prematura ou precoce tem consequências físicas e psicológicas; portanto, as mulheres podem precisar de apoio e acompanhamento médico de longo prazo.

**Métodos** Para correlacionar a dose de radiação ovariana com a função ovariana, foi usada a fórmula descrita por Wallace et al.: √g(z) = 10 ^(2-0,15z)^ , na qual “g(z)” e “z” representam a taxa de sobrevivência do oócito e a dose de radiação (em Gray), respectivamente. Ao simular diferentes idades e doses, observamos um padrão que poderia ser usado para simplificar a relação entre a dose de radiação e o tempo restante da função ovariana.

**Resultados** Obtivemos uma função linear entre a dose de radiação ovariana e a perda da função ovariana (LOF, na sigla em inglês) que é a porcentagem de diminuição no tempo até a falência ovariana em relação ao tempo esperado para uma mulher da mesma idade sem exposição à radiação. Para pacientes < 40 anos de idade e com doses de radiação ovariana < 5 Gy, a equação LOF = 2,70 + (11,08 x Dose) pode ser aplicada para estimar a redução no tempo até a insuficiência ovariana.

**Conclusão** O presente estudo relata um método teórico viável para estimar a perda da função ovariana. Estes achados podem melhorar potencialmente o manejo e o aconselhamento de pacientes jovens submetidas à radioterapia durante seus anos reprodutivos.

## Introduction

Radiation is an integral component of therapy for a variety of tumors that may affect young people. Most of these tumors are associated with high cure rates; therefore, treatment results in potential risk for survivorship issues. 1 2 3 For young women, premature ovarian insufficiency and decreased reproductive potential are important risks related to this treatment, with consequences regarding bone and cardiovascular health. 4 5 6 Total body craniospinal axis, whole abdominal, or pelvic irradiation potentially expose the ovaries to irradiation. 7 8 9 Radiotherapy is now a well-known cause of ovarian damage. The amount of injury is related to several variables, including the total radiation dose, the fractionation schedule, and age at the time of treatment. 10

The human ovary contains a limited number of primordial oocytes that reaches a peak at 5 months after conception and declines with increasing age in a biexponential fashion. This decline culminates in the menopause, when the number of oocytes is < 1,000, at an average age of 51 years old. 11 The ovaries are highly radiosensitive organs. Some authors have suggested that doses > 6 Gy in total body irradiation in young women induce premature ovarian insufficiency, whereas prepubertal women can tolerate even higher radiation doses. 2 In a large cohort of childhood cancer survivors, 215 cases (6.3%) developed premature ovarian insufficiency. Radiotherapy to the ovaries was the most significant risk factor for premature ovarian insufficiency, especially at doses ≥ 1,000 Gy, and exposure to the alkylating agents procarbazine and cyclophosphamide, at older ages. 12 Presumably, this reflects the number of oocytes at the time of exposure: a younger patient has more oocytes and, therefore, a wider fertility window.

Wallace et al. 13 created a mechanism to predict ovarian insufficiency according to the age of the patient and to the fractionated radiation dose received by the ovaries. In that model, they reported sterilizing dose of radiation for a known age at treatment and the age of ovarian insufficiency for total body radiation maximum dose of 3, 6, 9, and 12 Gy. This is the first model to predict with reliability the age at which ovarian insufficiency will supervene for any patient after treatment with a known dose of radiotherapy received by the ovaries. However, the described model does not allow an accessible evaluation of the decrease of the time for ovarian insufficiency for other doses and has limited use in clinical practice. Therefore, the present study aimed to review the model created by Wallace et al. and to develop a mathematical model to facilitate the prediction of ovarian insufficiency in the era of modern computed tomography (CT) radiotherapy planning.

## Methods

The present study was approved by the Ethics Committee of the involved institutions (CAAE number: 77681317.3.0000.5128). To correlate ovarian radiation dose with ovarian function, we used the formula described by Wallace et al.: 13 √g(z) = 10, 2 10 14 where “g(z)” and “z” represent oocyte survival rate and the radiation dose (in Gray), respectively. To solve the differential equation, we applied the fourth order of Runge-Kutta method using a Matlab algorithm and obtained the number of oocytes at a given age and calculated the residual number after irradiation at any dose. 14 The Runge–Kutta is an iterative method used in temporal discretization for the approximate solutions of ordinary differential equations. It has enabled a more accurate estimate of the radiosensitivity of the human oocyte. We considered 701,200 the initial number of oocytes at birth and 1,000 the number necessary at menopause. 15

For the statistical analysis, R software, version 3.4.2 (R Foundation, Vienna, Austria) was used. The loss of ovarian function (LOF) was defined as the percentage of decrease in the time to ovarian insufficiency compared with the time expected for a woman at the same age without irradiation exposition. Assuming that oocyte decay after irradiation was the same as that for nonirradiated oocytes, we simulated the remaining time for women aged between 10 and 50 years old for exposure to 0.5 Gy to doses that cause immediate ovarian insufficiency. From these remaining times, we could obtain a simple equation relating dose and time until menopause.

To define the relationship between loss of ovarian function and dose, a linear regression was fitted. 16 To verify the performance of the model, a cross-validation was performed with the leave-one-out method. At the end of the cross-validation process, the root-mean-square error (RMSE) was calculated.

## Results

**Fig. 1:**
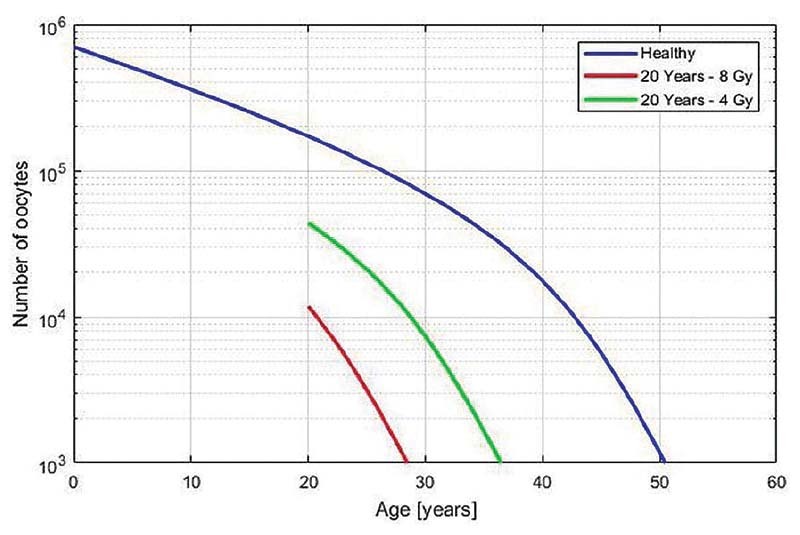
The influence of aging and fractionated radiation on oocyte number. Blue curve: healthy women; Green curve: woman who received 4 Gy in ovaries at 20 years old; Red curve: woman who received 8 Gy in ovaries at 20 years old.

The numerical solution of the differential equation obtained from the Matlab algorithm is shown in Figure 1 . The blue curve represents the number of oocytes in a healthy woman at a given age. The green curve shows oocyte decay in a 20-year-old patient who received a 4-Gy dose to the ovaries. The red curve shows the same simulation with an 8-Gy dose. From these simulations, it is clear that the radiation dose greatly affects the remaining time until ovarian insufficiency.

**Fig. 2:**
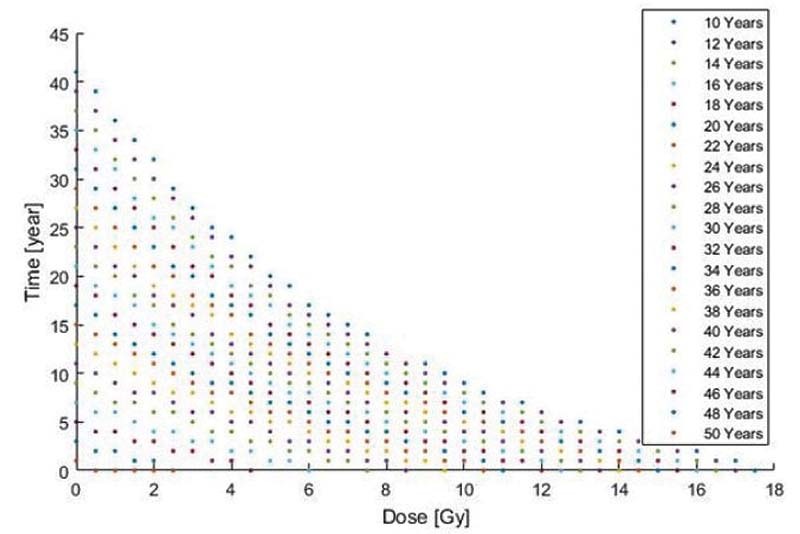
Expected time of ovarian function remaining after a certain radiation dose for different ages.

**Fig. 3:**
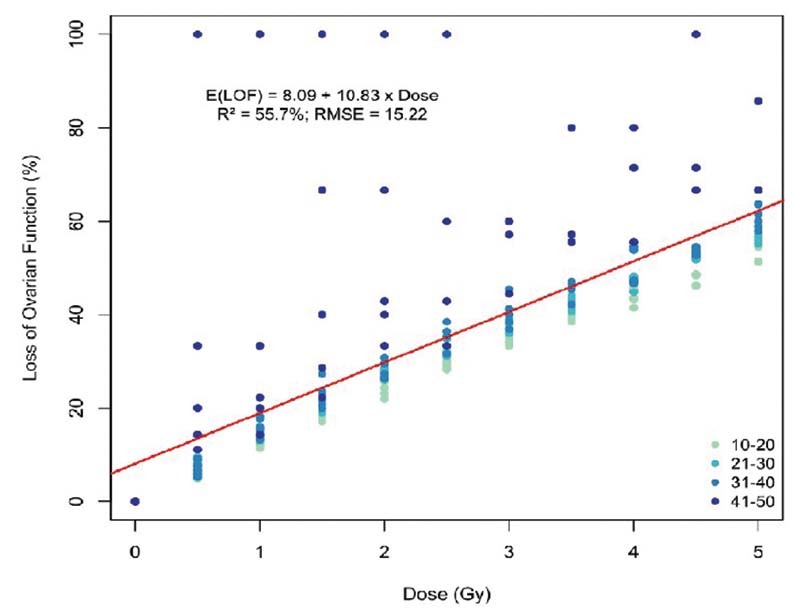
Relationship between loss of ovarian function and ovarian radiation dose for the complete database.

**Fig. 4:**
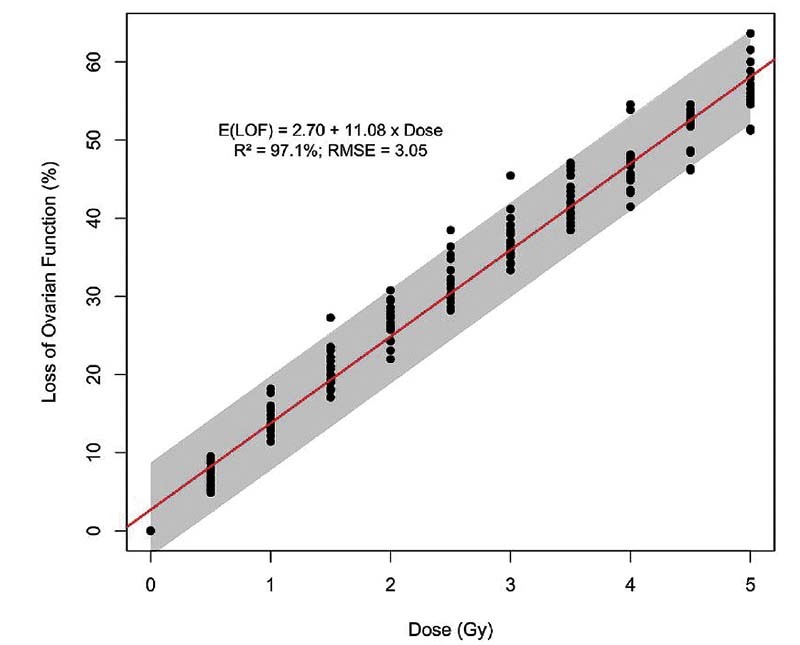
Relationship between the loss of ovarian function according to the radiation dose in women aged 10 to 40 years.

Figure 2 shows the remaining time of ovarian function for ages between 10 and 50 years old for doses ranging from zero to those that cause ovarian insufficiency. The first proposed model ( Figure 3 ) included the entire database but did not represent a satisfactory adjustment (RMSE = 15.22; R ^2^ = 55.7%), as the ovarian function loss for women > 40 years old revealed a distinct pattern. Therefore, we readjusted the model excluding women > 40 years old. This new model, represented in Figure 4 , obtained the equation LOF = 2.70 + (11.08 x Dose) with an RMSE of 3.05, indicating that the value generated by the formula can range from - 6 to + 6 years (3.05 × 1.96) with a 95% confidence interval (CI).

## Discussion

The equation proposed in the present study can be considered a readily accessible way to predict ovarian insufficiency after radiotherapy with a known dose received by the ovaries. For example, if the ovaries were exposed to 4 Gy at age 27, the patient would have a decrease in time to ovarian insufficiency of 47% [2.7 + (11.08 × 4)] to what would be expected in women of the same age without radiation exposure. In other words, considering 51 years old as the median age of natural menopause, if a 27-year-old patient was exposed to 4 Gy, she would lose 11 years of ovarian function, and would enter menopause at 40 years old.

With no biochemical markers available to predict premature ovarian insufficiency, such a model that determines the extent of radiotherapy-induced damage and allows an assessment of the “fertile window” will have a significant impact on reproductive counseling for young women with cancer. For those young women who are at risk of a very early menopause, it is possible to consider counseling them on the options currently available to preserve their fertility before their treatment starts. Making decisions about preserving future fertility requires that patients receive information from their doctors.

**Fig. 5:**
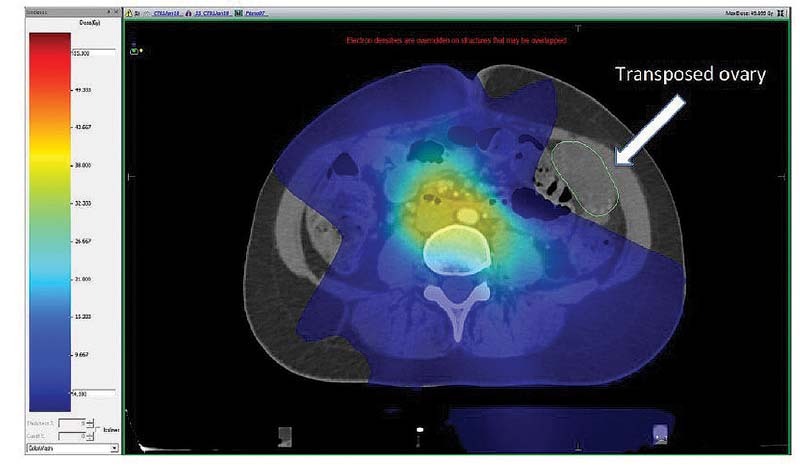
Computed tomography planning with volumetric arc therapy (VMAT) (Monaco TPS – version 5.51) of a 27-year-old patient in adjuvant radiation for cervical cancer. Isodose (blue color wash) of 4 Gy does not touch the transposed ovary (white arrow).

Limitation of radiation dose to the ovary is practiced in adult women with cervical cancer in childbearing age submitted to adjuvant radiotherapy. In the era of radiotherapy, treatment planning based on CT and sophisticated external beam irradiation techniques, such as intensity modulated radiotherapy (IMRT) and volumetric modulated arc radiotherapy (VMAT), sharp dose gradients against normal tissue with a considerable reduction of ovarian radiation dose are possible. 17 In order to minimize the effects of induced menopause, ovarian transposition can be surgically performed and modern radiation techniques can spare the ovaries from high radiations doses ( Figure 5 ). 18 19 20 Calculation of the dose of radiation received by each ovary, combined with a more accurate estimate of the radiosensitivity of the human oocyte, could facilitate our ability to provide more scientific fertility counseling to young women at risk of premature menopause following the successful treatment of cancer. Wallace et al. 13 reported the first model to predict the age of ovarian insufficiency after treatment with a known dose of radiotherapy. In their publication, they provided a table with the predicted age of onset of ovarian insufficiency for ages of treatment from 0 to 30 years old for fixed doses of 3, 6, 9, and 12 Gy. 13 Our mathematical model has a sharp CI and yields similar results to those of the table developed by Wallace et al. We observed by our model that the pattern of oocyte/ovarian function loss occurs with different patterns for women younger or older than 40 years old. This can be explained by an increased rate of oocyte loss that occurs around the age of 37 years old, when ∼ 25,000 follicles remain. 21 22

It is important to note that we did not consider the ovaries receiving different doses, and the results can only be applied to both ovaries receiving the same radiation dose. We acknowledge that this is a predictive model based on preclinical work and that it does not take into account the current use of combined modality treatments. The results do not contemplate the chemotherapy impact on oocytes damage. Radiotherapy is frequently used in combination with chemotherapy for the treatment of cancer. Potentially gonadotoxic chemotherapy may be a contributory factor to the development of premature menopause. It is also important to consider the effect of radiation towards the uterus in terms of fertility. Radiation towards the uterus reduces the size of the organ, makes it less elastic, and, therefore, enhances the risk of spontaneous abortion and premature birth. 3 4 23

## Conclusion

In summary, the present study enables counseling women on their reproductive potential following the successful treatment of their cancer. We have constructed a mathematical model that could be used to quickly estimate ovarian insufficiency after radiotherapy. More studies with clinical outcomes and follow-up of the patients are needed to validate and optimize the proposed model.
